# Dynamics of the Brain Functional Network Associated With Subjective Cognitive Decline and Its Relationship to Apolipoprotein E €4 Alleles

**DOI:** 10.3389/fnagi.2022.806032

**Published:** 2022-03-09

**Authors:** Baiwan Zhou, Xiaojia Wu, Lin Tang, Chuanming Li

**Affiliations:** Department of Radiology, The Second Affiliated Hospital of Chongqing Medical University, Chongqing, China

**Keywords:** subjective cognitive decline, resting-state, neuroimaging, dynamic functional connectome, static functional connectome

## Abstract

The aim of our study was to explore the dynamic functional alterations in the brain in patients with subjective cognitive decline (SCD) and their relationship to apolipoprotein E (APOE) €4 alleles. In total, 95 SCD patients and 49 healthy controls (HC) underwent resting-state functional magnetic resonance imaging (rs-fMRI). Then, the mean time series of 90 cortical or subcortical regions were extracted based on anatomical automatic labeling (AAL) atlas from the preprocessed rs-fMRI data. The static functional connectome (SFC) and dynamic functional connectome (DFC) were constructed and compared using graph theory methods and leading eigenvector dynamics analysis (LEiDA), respectively. The SCD group displayed a shorter lifetime (*p* = 0.003, false discovery rate corrected) and lower probability (*p* = 0.009, false discovery rate corrected) than the HC group in a characteristic dynamic functional network mainly involving the bilateral insular and temporal neocortex. No significant differences in the SFC were detected between the two groups. Moreover, the lower probability in the SCD group was found to be negatively correlated with the number of APOE ε4 alleles (*r* = −0.225, *p* = 0.041) in a partial correlation analysis with years of education as a covariate. Our results suggest that the DFC may be a more sensitive parameter than the SFC and can be used as a potential biomarker for the early detection of SCD.

## Introduction

Alzheimer’s disease (AD) is the most common neurodegenerative cause of dementia and is associated with significant morbidity and mortality. AD has a heavy economic burden on the health care system (Oboudiyat et al., 2013). Subjective cognitive decline (SCD), also known as significant memory concern (SMC), has been suggested to be the preclinical stage of AD, which is characterized by a subjective decline in cognitive function without any notable alterations in neuropsychological test results (Sperling et al., 2011; Viviano and Damoiseaux, 2020). Pathophysiological changes in the brain have been proved to occur long before cognitive symptoms (Morris, 2005). Therefore, exploring biomarkers of SCD will contribute to the early diagnosis of AD. Early diagnosis and timely clinical intervention can greatly improve the prognosis of the patients. As a non-invasive neuroimaging technique, resting-state functional magnetic resonance imaging (rs-fMRI) has been widely applied to explore the neural mechanisms underlying SCD based on various neuroimaging measures, such as regional homogeneity (Li et al., 2021), amplitude of low-frequency fluctuations (Sun et al., 2016), functional connectivity (Hafkemeijer et al., 2013; Dillen et al., 2016) and complex network measures (Chen et al., 2020; Xue et al., 2020).

However, previous studies have mainly focused on static assessments and ignored the dynamic alterations in brain activity, while recent studies have shown that brain activity changes over time and that dynamic characteristics can provide useful information for understanding brain cognitive functions (Chang and Glover, 2010; Hutchison et al., 2013; Preti et al., 2017). Regarding SCD, recent studies have attempted to explore changes in the dynamic functional connectome (DFC) by using the sliding window approach (Sakoglu et al., 2010). Xie et al. found that centrality frequency in anterior cortical regions, especially in the default mode network (DMN), weakened its contribution to cognitive performance (Xie et al., 2019). Chen et al. (2021) observed increased fractional windows and mean dwell time in a hyper-connected state and a reduced number of state transitions in the SCD group compared to the healthy control (HC) group. However, the sliding window approach is limited by the choice of window length, which may affect the temporal resolution as well as statistical validation (Hindriks et al., 2016; Preti et al., 2017). Thus, a data-driven phase coherence technique, leading eigenvector dynamics analysis (LEiDA; Cabral et al., 2017), has been developed to overcome these limitations; LEiDA does not require any thresholding and is sensitive to phase-shifted patterns (Glerean et al., 2012; Cabral et al., 2017). To date, no study has used LEiDA to investigate alterations in the DFC in SCD patients.

In this study, we conducted an rs-fMRI study by using LEiDA to explore alterations in the DFC related to SCD, while the static functional connectome (SFC) was also evaluated based on graph theory methods. Furthermore, a previous study reported that the number of apolipoprotein E (APOE) €4 alleles was related to severe memory loss in AD (Lehtovirta et al., 1996), but whether the number of alleles is associated with alterations in the DFC in the context of SCD remains unclear. Thus, the relationship between the number of APOE €4 alleles and altered DFC parameters was also evaluated to explore the link between brain function and genetics in SCD.

## Materials and Methods

### Participants

All the data in this study were downloaded from the Alzheimer’s Disease Neuroimaging Initiative (ADNI) database^1^, and informed consent was obtained in accordance with the Declaration of Helsinki. Participants were included if they were diagnosed with SCD according to the standard criteria described in the ADNI-2 procedures manual^2^. The key inclusion criterion was a self-reported cognitive decline from the participant without impairment on the Logical Memory II subscale (delayed paragraph recall, paragraph A only) from the Wechsler Memory Scale-Revised. The HC showed no signs of depression, mild cognitive impairment (MCI), dementia or self-reported cognitive decline. APOE genotyping was performed at the time of participant enrollment. For more details please see the reference paper (Saykin et al., 2010). To maintain consistency of the scan parameters, only participants scanned by the Magnetom Prisma 3T scanner were included. Thus, a total of 95 SCD patients and 49 HC were included. Detailed information is shown in Table 1.

**TABLE 1:** Demographic and clinical data^a^.

Variables	SCD	HC	*P* value^b^
Sample size	95	49	–
Age (years)^c^	70.69 ± 6.37	71.33 ± 6.16	0.945
Sex (M/F)	32/63	17/32	1.000
Education (years)	16.96 ± 2.21	17.00 ± 1.78	0.907
ADAS11	9.28 ± 2.50	8.79 ± 2.49	0.689
ADAS13	13.48 ± 4.1	12.0 ± 4.10	0.444
MMSE	29.07 ± 1.12	28.92 ± 1.13	0.585
RAVLT_immediate	45.47 ± 10.75	47.71 ± 9.32	0.531
RAVLT_learning	6.09 ± 2.19	6.45 ± 2.42	0.626
RAVLT_forgetting	3.97 ± 3.07	1.90 ± 4.39	0.815
RAVLT_perc_forgetting	37.07 ± 30.36	19.73 ± 41.86	0.372
LMDRT	12.81 ± 3.95	13.94 ± 3.29	0.117
MoCA	25.95 ± 2.70	26.35 ± 2.41	0.386
APOE (0, 1, 2)	52, 27, 5	31, 10, 2	0.519

*^a^Data are presented as the mean ± standard deviation.*

*^b^P values for sex and APOE were obtained by using chi-square tests, and p values for the other variables were obtained by using two-sample t-tests.*

*^c^Age was defined at the time of MRI scanning.*

### Resting-State Functional Magnetic Resonance Imaging Data Acquisition and Preprocessing

Resting-state functional magnetic resonance imaging data were obtained from a 3T MRI scanner (Magnetom Prisma, Siemens, Erlangen, Germany). When the participants were scanned, they were asked to keep their eyes open and to stay awake. Each examination lasted for 591 s and contained 197 image volumes. The following parameters were used: field strength = 3.0 Tesla; flip angle = 90.0 degrees; manufacturer = SIEMENS; matrix X = 448.0 pixels; matrix Y = 448.0 pixels; mfg model = Prisma_fit; pixel spacing X = 3.4 mm; pixel spacing Y = 3.4 mm; pulse sequence = EP; slices = 197.0; slice thickness = 3.4 mm; echo time (TE) = 30.0 ms; and repetition time (TR) = 3000.0 ms.

Data preprocessing was performed using Gretna version 2.0 software^3^. First, the initial 10 time points were deleted to minimize the impact of signal instability at the beginning of the MRI scan, and corrections were carried out for the acquisition delay between slices. Friston 24-parameter correction was used to ensure that the effects of head motion did not contribute to the results we obtained. Then, images were normalized to the echo-planar imaging (EPI) template with a voxel size of 3 × 3 × 3 mm and smoothed by using a 4-mm full-width at half-maximum (FWHM) Gaussian kernel. Then the images were linearly detrended. The white matter signals and cerebral spinal fluid signals were also regressed out. Finally, a filter (bandpass: 0.01–0.1 Hz) was used to minimize the influences of low-frequency drift and high-frequency noise. Head movement with an average FD > 0.25 was considered excessive. None of the subject exceeded this threshold.

### Static Functional Connectome Analysis

This step was performed by using Gretna version 2.0 software. First, the whole brain was separated into 90 cortical or subcortical regions based on the anatomical automatic labeling (AAL) atlas (Fenchel et al., 2008), and the mean time series of these regions were extracted. Pearson’s correlation analysis and Fisher r-to-Z transformation were performed to obtain a 90 × 90 undirected and weighted correlation matrix for each subject. Finally, two network efficiency parameters (Latora and Marchiori, 2001), local efficiency (E_loc_) and global efficiency (E_glob_), as well as two nodal parameters, nodal degree (Rubinov and Sporns, 2010) and nodal efficiency (Achard and Bullmore, 2007), were calculated to investigate the topology of the SFC, while functional connectivity was evaluated by using the network-based statistics (NBS) method (Zalesky et al., 2010). For additional details, see the Supplementary Material.

### Dynamic Functional Connectivity Analysis

The DFC analysis was performed by using LEiDA (Cabral et al., 2017). Briefly, first, the instantaneous blood oxygenation level-dependent (BOLD) synchronization matrix was calculated by using the Hilbert transform based on the mean time series of the 90 brain regions for each subject, and the dominant dynamic pattern of each time point was identified as the leading eigenvector. Then, the leading eigenvectors were clustered to different phase-locked (PL) states by applying a k-means clustering algorithm. This method usually requires researchers to choose a number of clusters (k). In this study, the algorithm was run for 10 iterations with the value of k between 3 and 12, with higher k values resulting in more fine-grained configurations (Alonso Martinez et al., 2020). For each state of each iteration, the probability was calculated as the number of times each state was dominant, and the lifetime (LT) was calculated as the average duration during which a state was dominant, in seconds. To visually identify each PL state, these results were plotted in the cortex by using BrainNet Viewer^4^, and the characteristic leading eigenvectors for each PL state were identified as the leading eigenvectors that were detected positively only in this PL state rather than every other PL state. For additional details, see the reference paper (Cabral et al., 2017).

### Correlation Analysis

To explore the relationship between changed DFC parameters (LT and probability of PL state 3; *k* = 4) and the number of APOE ε4 alleles in the SCD group, correlation analysis was performed by using partial correlation analysis with years of education as a covariate.

### Statistical Analysis

For the demographic, clinical and neuropsychological data, the group differences in sex and the number of APOE ε4 alleles were evaluated by using chi-square tests, while the group differences in other variables were all evaluated by using two-sample *t*-tests with an alpha threshold of 0.05.

The SFC and DFC properties between the SCD group and HC group were compared by using two-sample *t*-tests with a false discovery rate (FDR)-corrected alpha threshold of 0.01.

## Results

### Demographic, Clinical and Neuropsychological Data

Regarding the demographic data, no significant differences in age, sex or years of education were found between the two groups. Regarding the neuropsychological data, no significant differences were found in any of the neuropsychological test results. Detailed information is shown in Table 1.

### Dynamic Functional Connectome Analysis

**FIGURE 1:**
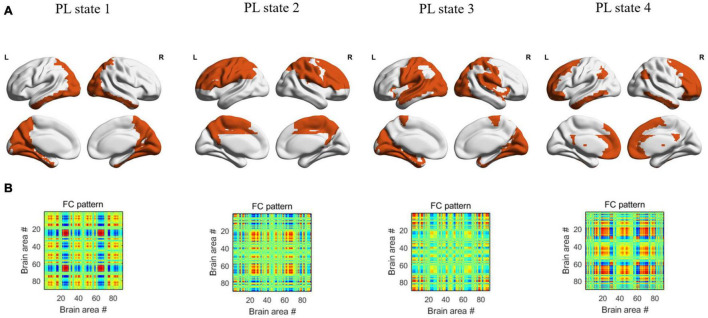
The patterns of 4 PL states detected by clustering the set of leading eigenvectors into 4 clusters. **(A)** Cortical space representation of each PL state, the regions colored with orange represent the leading eigenvectors with positive sign, while the regions colored with white represent the leading eigenvectors with negative sign, for each PL state. **(B)** The 90×90 connectivity pattern corresponding to each state.

**FIGURE 2:**
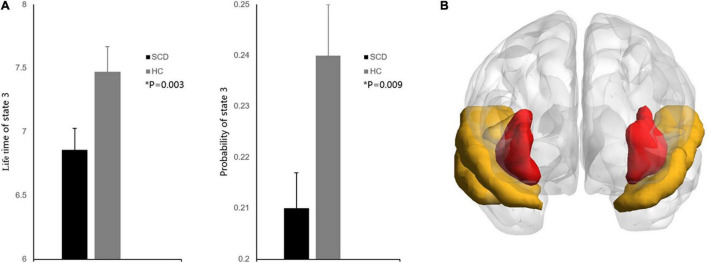
**(A)** Group comparisons of state probability and LT between the SCD and HC groups. Bar plot representing the group differences between the SCD and HC groups. Asterisks indicate significant group differences between the two groups after false discovery rate correction (*P* < 0.01). Error bars represent standard error. **(B)** The characteristic regions for PL state 3. Regions colored red represent the bilateral insular, regions colored yellow represent the bilateral temporal neocortex.

In the dynamic functional analysis, a significant difference was detected only in PL state 3 when the DFC was divided into four PL states (Figure 1). There was a shorter LT (*p* = 0.003, FDR corrected) and a lower probability (*p* = 0.009, FDR corrected) in the SCD group than in the HC group (Figure 2A) (see Supplementary Material for all *p* values for all partition models and leading eigenvectors for each PL state). The characteristic leading eigenvectors of PL state 3 mainly included the bilateral insular and temporal neocortex (Figure 2B).

### Static Functional Connectome Analysis

In the SFC analysis, no significant differences in topological parameters or functional connectivity were found between the SCD group and HC group after FDR correction.

### Correlation Analysis

**FIGURE 3:**
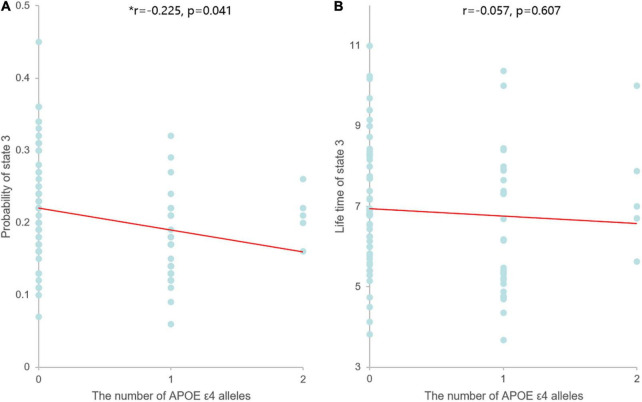
Scatter plots of the correlation between the dynamic measures of PL state 3 and the number of APOE ε4 alleles. Asterisks indicate significant correlation between the two parameters. **(A)** The probability of PL state 3 was negatively correlated with the number of APOE ε4 alleles, and linear model fitting is shown over the scatterplot (red line). **(B)** The LT of PL state 3 was not significantly correlated with the number of APOE ε4 alleles, and linear model fitting is shown over the scatterplot (red line).

In our correlation analysis, the probability of PL state 3 was negatively correlated with the number of APOE ε4 alleles (*r* = −0.225, *p* = 0.041), while the LT of PL state 3 was not significantly correlated with the number of APOE ε4 alleles (*r* = −0.057, *p* = 0.607) (Figure 3).

## Discussion

In this study, we evaluated both static and dynamic alterations in the brain functional network in the SCD group compared with the HC group. Our dynamic analysis revealed a shorter LT and lower probability of occurrence in PL state 3 in the SCD group than the HC group. Our results suggested that shorter LT and lower probability in PL state 3 are characteristics and biological markers of SCD, which may have great potential in clinical diagnosis. LT is an indicator of the average duration of time in one PL state, and the probability indicates the number of times that one PL state was dominant. Previously, alterations in LT and probability have been found to be related to cognitive performance in several functional networks (Cabral et al., 2017). In our study, the characteristic region associated with PL state 3 consisted of the bilateral insular and temporal neocortex. The temporal neocortex is considered to participate in the formation of verbal memory (Kucewicz et al., 2018), auditory memory (Munoz-Lopez et al., 2015) and visual recognition memory (Boggio et al., 2009), as well as in the regulation of memory retrieval (Vaz et al., 2019). While the insula has not been directly correlated with memory function, it was considered to play a role in controlling engagement of the DMN and executive control network (CEN; Menon and Uddin, 2010; Hu et al., 2017), which are independently related to episodic memory (Buckner et al., 2008) and working memory (Seeley et al., 2007). Thus, we can speculate that SCD may be related to dynamic functional alterations in characteristic regions, including the insular and temporal neocortex, which are not only involved in memory functions but also associated with the ability to properly recruit different regions.

In contrast to the remarkable dynamic functional alterations, no significant group differences were detected in our static analysis, including the topological parameters and static functional connectivity. These results suggested that the DFC is more sensitive than the SFC and can reflect an earlier stage of brain dysfunction associated with cognitive impairment. One possible reason is that the DFC describes brain activity at each time point, while the SFC describes the average state of brain activity over a period of time. The average value will mask small differences between internal data, resulting in reduced sensitivity. In this study, LEiDA was used for the DFC analysis. As the preclinical stage of AD, alterations of brain function in SCD patients may be very slight. Compared with other DFC analysis methods (such as sliding window method), LEiDA has higher time resolution. Small changes in brain function in patients with SCD could be found. A previous study also reported that no significant differences in whole-brain BOLD signal standard deviation were detected between the SCD and normal groups (Scarapicchia et al., 2019). In addition, some studies also revealed no group differences in the static analysis of topological parameters between AD dementia patients and normal controls (NCs; Peraza et al., 2015; Schumacher et al., 2019).

In the correlation analysis, we found that the probability of PL state 3 was significantly correlated with the number of APOE ε4 alleles. This is the first study to identify the relationship between the SCD dynamic network and genes, which provides further evidence that changes in the DFC can reflect related neural changes and are a genetic feature. APOE ε4 alleles have been shown to be associated with several forms of impaired neuronal maintenance (Haan et al., 1999), such as the development of β-amyloid and neurofibrillary tangles (Schmechel et al., 1993; Gomez-Isla et al., 1996). Thus, increased APOE ε4 alleles may bring about functional impairment, which may lead to alterations in dynamic brain function. Previous studies have also found that APOE ε4 alleles were associated with structural and functional alterations in the brain in the prodromal stage of AD (Filippini et al., 2009a,b; Brown et al., 2011).

Several limitations should be noted. First, in this study, we observed dynamic functional alterations in the brain in the prodromal stage of AD; however, it is still not clear whether these alterations will further develop with the progression of disease, so longitudinal research should be considered in future studies. In addition, each rs-fMRI examination lasted 591 s in our study, which is slightly shorter than the 10 min that some researchers have suggested, but it was still enough to describe the dynamics in brain function. Moreover, in the preprocessing of the rs-fMRI data, we did not regress the global signal because of the rich information it contains (Li et al., 2019), as the previous study did (Cabral et al., 2017). However, this made the influence of noise more marked (Murphy and Fox, 2017), so whether to regress the global signal needs to be further discussed. Finally, LEiDA is very susceptible to the influence of time points. Future studies should focus on the difference of DFC between the data with different time points.

In this study, we investigated the differences in both the SFC and DFC between those with SCD and HC, and we observed a significant difference only in the DFC and not in the SFC, which suggested that the DFC may be a more informative parameter than the SFC measures. Moreover, the DFC parameters were found to be negatively related to the number of APOE ε4 alleles, which provides further evidence that dynamic alterations in brain function may be associated with genetics and therefore serve as a potential biomarker for the early detection of SCD.

## Data Availability Statement

The data used in this study were downloaded from the Alzheimer’s Disease Neuroimaging Initiative (ADNI) website (adni.loni.usc.edu), the data was collected in different institution. And the ADNI study was approved by an ethics committee on human experimentation at each institution, and written informed consent was obtained from all participants.

## Ethics Statement

Ethical review and approval was not required for the study on human participants because all the data in this study were downloaded from the Alzheimer’s Disease Neuroimaging Initiative (ADNI) database.

## Author Contributions

BZ and CL designed the study and wrote the manuscript. XW and LT collected and processed the data. CL revised the manuscript. All authors reviewed the manuscript and approved the final version to be published.

## Conflict of Interest

The authors declare that the research was conducted in the absence of any commercial or financial relationships that could be construed as a potential conflict of interest.

## Publisher’s Note

All claims expressed in this article are solely those of the authors and do not necessarily represent those of their affiliated organizations, or those of the publisher, the editors and the reviewers. Any product that may be evaluated in this article, or claim that may be made by its manufacturer, is not guaranteed or endorsed by the publisher.

## Footnotes

1
http://adni.loni.usc.edu

2
http://www.adni-info.org

3
http://www.nitrc.org/projects/gretna/

4
http://www.nitrc.org/projects/bnv/

## Abbreviations

SCD: subjective cognitive decline
HCs: healthy controls
CDSB: clinical dementia rating scale-sum of boxes
F: female
M: male
MMSE: mini-mental state exam
MoCA: montreal cognitive assessment
RAVLT: rey auditory verbal learning test
LMDRT: = logical memory – delayed recall-total number of story units recalled
ADAS: Alzheimer’s disease assessment scale
FAQ: functional activities questionnaire
APOE: the number of APOE ε 4 alleles.

## References

[B1] Achard S. Bullmore E. (2007). Efficiency and cost of economical brain functional networks. *PLoS Comput. Biol.* 3:e17. 10.1371/journal.pcbi.0030017 17274684 PMC1794324

[B2] Alonso Martinez S. Deco G. Ter Horst G. J. Cabral J. (2020). The dynamics of functional brain networks associated with depressive symptoms in a nonclinical sample. *Front. Neural Circuits* 14:570583. 10.3389/fncir.2020.570583 33071760 PMC7530893

[B3] Boggio P. S. Khoury L. P. Martins D. C. Martins O. E. de Macedo E. C. Fregni F. (2009). Temporal cortex direct current stimulation enhances performance on a visual recognition memory task in Alzheimer disease. *J. Neurol. Neurosurg. Psychiatry* 80 444–447. 10.1136/jnnp.2007.141853 18977813

[B4] Brown J. A. Terashima K. H. Burggren A. C. Ercoli L. M. Miller K. J. Small G. W. (2011). Brain network local interconnectivity loss in aging APOE-4 allele carriers. *Proc. Natl. Acad. Sci. U.S.A.* 108 20760–20765. 10.1073/pnas.1109038108 22106308 PMC3251140

[B5] Buckner R. L. Andrews-Hanna J. R. Schacter D. L. (2008). The brain’s default network: anatomy, function, and relevance to disease. *Ann. N. Y. Acad. Sci.* 1124 1–38. 10.1196/annals.1440.011 18400922

[B6] Cabral J. Vidaurre D. Marques P. Magalhaes R. Silva Moreira P. Miguel Soares J. (2017). Cognitive performance in healthy older adults relates to spontaneous switching between states of functional connectivity during rest. *Sci. Rep.* 7:5135. 10.1038/s41598-017-05425-7 28698644 PMC5506029

[B7] Chang C. Glover G. H. (2010). Time-frequency dynamics of resting-state brain connectivity measured with fMRI. *Neuroimage* 50 81–98. 10.1016/j.neuroimage.2009.12.011 20006716 PMC2827259

[B8] Chen H. Sheng X. Luo C. Qin R. Ye Q. Zhao H. (2020). The compensatory phenomenon of the functional connectome related to pathological biomarkers in individuals with subjective cognitive decline. *Transl. Neurodegener.* 9:21. 10.1186/s40035-020-00201-6 32460888 PMC7254770

[B9] Chen Q. Lu J. Zhang X. Sun Y. Chen W. Li X. (2021). Alterations in dynamic functional connectivity in individuals with subjective cognitive decline. *Front. Aging Neurosci.* 13:646017. 10.3389/fnagi.2021.646017 33613274 PMC7886811

[B10] Dillen K. N. H. Jacobs H. I. L. Kukolja J. von Reutern B. Richter N. Onur O. A. (2016). Aberrant functional connectivity differentiates retrosplenial cortex from posterior cingulate cortex in prodromal Alzheimer’s disease. *Neurobiol. Aging* 44 114–126. 10.1016/j.neurobiolaging.2016.04.010 27318139

[B11] Fenchel M. Thesen S. Schilling A. (2008). Automatic labeling of anatomical structures in MR fastview images using a statistical atlas. *Med. Image Comput. Comput. Assist. Interv.* 11(Pt 1) 576–584. 10.1007/978-3-540-85988-8_69 18979793

[B12] Filippini N. MacIntosh B. J. Hough M. G. Goodwin G. M. Frisoni G. B. Smith S. M. (2009a). Distinct patterns of brain activity in young carriers of the APOE-epsilon4 allele. *Proc. Natl. Acad. Sci. U.S.A.* 106 7209–7214. 10.1073/pnas.0811879106 19357304 PMC2678478

[B13] Filippini N. Zarei M. Beckmann C. F. Galluzzi S. Borsci G. Testa C. (2009b). Regional atrophy of transcallosal prefrontal connections in cognitively normal APOE epsilon4 carriers. *J. Magn. Reson. Imaging* 29 1021–1026. 10.1002/jmri.21757 19388128

[B14] Glerean E. Salmi J. Lahnakoski J. M. Jaaskelainen I. P. Sams M. (2012). Functional magnetic resonance imaging phase synchronization as a measure of dynamic functional connectivity. *Brain Connect.* 2 91–101. 10.1089/brain.2011.0068 22559794 PMC3624768

[B15] Gomez-Isla T. West H. L. Rebeck G. W. Harr S. D. Growdon J. H. Locascio J. J. (1996). Clinical and pathological correlates of apolipoprotein E epsilon 4 in Alzheimer’s disease. *Ann. Neurol.* 39 62–70. 10.1002/ana.410390110 8572669

[B16] Haan M. N. Shemanski L. Jagust W. J. Manolio T. A. Kuller L. (1999). The role of APOE epsilon4 in modulating effects of other risk factors for cognitive decline in elderly persons. *JAMA* 282 40–46. 10.1001/jama.282.1.40 10404910

[B17] Hafkemeijer A. Altmann-Schneider I. Oleksik A. M. van de Wiel L. Middelkoop H. A. van Buchem M. A. (2013). Increased functional connectivity and brain atrophy in elderly with subjective memory complaints. *Brain Connect.* 3 353–362. 10.1089/brain.2013.0144 23627661 PMC3749691

[B18] Hindriks R. Adhikari M. H. Murayama Y. Ganzetti M. Mantini D. Logothetis N. K. (2016). Can sliding-window correlations reveal dynamic functional connectivity in resting-state fMRI? *Neuroimage* 127 242–256. 10.1016/j.neuroimage.2015.11.055 26631813 PMC4758830

[B19] Hu X. Uhle F. Fliessbach K. Wagner M. Han Y. Weber B. (2017). Reduced future-oriented decision making in individuals with subjective cognitive decline: a functional MRI study. *Alzheimers Dement. (Amst.)* 6 222–231. 10.1016/j.dadm.2017.02.005 28393099 PMC5376255

[B20] Hutchison R. M. Womelsdorf T. Allen E. A. Bandettini P. A. Calhoun V. D. Corbetta M. (2013). Dynamic functional connectivity: promise, issues, and interpretations. *Neuroimage* 80 360–378. 10.1016/j.neuroimage.2013.05.079 23707587 PMC3807588

[B21] Kucewicz M. T. Berry B. M. Miller L. R. Khadjevand F. Ezzyat Y. Stein J. M. (2018). Evidence for verbal memory enhancement with electrical brain stimulation in the lateral temporal cortex. *Brain* 141 971–978. 10.1093/brain/awx373 29324988

[B22] Latora V. Marchiori M. (2001). Efficient behavior of small-world networks. *Phys. Rev. Lett.* 87:198701. 10.1103/PhysRevLett.87.198701 11690461

[B23] Lehtovirta M. Soininen H. Helisalmi S. Mannermaa A. Helkala E. L. Hartikainen P. (1996). Clinical and neuropsychological characteristics in familial and sporadic Alzheimer’s disease: relation to apolipoprotein E polymorphism. *Neurology* 46 413–419. 10.1212/wnl.46.2.413 8614504

[B24] Li J. Bolt T. Bzdok D. Nomi J. S. Yeo B. T. T. Spreng R. N. (2019). Topography and behavioral relevance of the global signal in the human brain. *Sci. Rep.* 9:14286. 10.1038/s41598-019-50750-8 31582792 PMC6776616

[B25] Li S. Daamen M. Scheef L. Gaertner F. C. Buchert R. Buchmann M. (2021). Abnormal regional and global connectivity measures in subjective cognitive decline depending on cerebral amyloid status. *J. Alzheimers Dis.* 79 493–509. 10.3233/JAD-200472 33337359

[B26] Menon V. Uddin L. Q. (2010). Saliency, switching, attention and control: a network model of insula function. *Brain Struct. Funct.* 214 655–667. 10.1007/s00429-010-0262-0 20512370 PMC2899886

[B27] Morris J. C. (2005). Early-stage and preclinical Alzheimer disease. *Alzheimer Dis. Assoc. Disord.* 19 163–165. 10.1097/01.wad.0000184005.22611.cc 16118535

[B28] Munoz-Lopez M. Insausti R. Mohedano-Moriano A. Mishkin M. Saunders R. C. (2015). Anatomical pathways for auditory memory II: information from rostral superior temporal gyrus to dorsolateral temporal pole and medial temporal cortex. *Front. Neurosci.* 9:158. 10.3389/fnins.2015.00158 26041980 PMC4435056

[B29] Murphy K. Fox M. D. (2017). Towards a consensus regarding global signal regression for resting state functional connectivity MRI. *Neuroimage* 154 169–173. 10.1016/j.neuroimage.2016.11.052 27888059 PMC5489207

[B30] Oboudiyat C. Glazer H. Seifan A. Greer C. Isaacson R. S. (2013). Alzheimer’s disease. *Semin. Neurol.* 33 313–329. 10.1055/s-0033-1359319 24234352

[B31] Peraza L. R. Taylor J. P. Kaiser M. (2015). Divergent brain functional network alterations in dementia with Lewy bodies and Alzheimer’s disease. *Neurobiol. Aging* 36 2458–2467. 10.1016/j.neurobiolaging.2015.05.015 26115566 PMC4706129

[B32] Preti M. G. Bolton T. A. Van De Ville D. (2017). The dynamic functional connectome: state-of-the-art and perspectives. *Neuroimage* 160 41–54. 10.1016/j.neuroimage.2016.12.061 28034766

[B33] Rubinov M. Sporns O. (2010). Complex network measures of brain connectivity: uses and interpretations. *Neuroimage* 52 1059–1069. 10.1016/j.neuroimage.2009.10.003 19819337

[B34] Sakoglu U. Pearlson G. D. Kiehl K. A. Wang Y. M. Michael A. M. Calhoun V. D. (2010). A method for evaluating dynamic functional network connectivity and task-modulation: application to schizophrenia. *MAGMA* 23 351–366. 10.1007/s10334-010-0197-8 20162320 PMC2891285

[B35] Saykin A. J. Shen L. Foroud T. M. Potkin S. G. Swaminathan S. Kim S. (2010). Alzheimer’s disease neuroimaging initiative biomarkers as quantitative phenotypes: genetics core aims, progress, and plans. *Alzheimers Dement.* 6 265–273. 10.1016/j.jalz.2010.03.013 20451875 PMC2868595

[B36] Scarapicchia V. Garcia-Barrera M. MacDonald S. Gawryluk J. R. (2019). Resting state BOLD variability is linked to white matter vascular burden in healthy aging but not in older adults with subjective cognitive decline. *Front. Hum. Neurosci.* 13:429. 10.3389/fnhum.2019.00429 31920589 PMC6936515

[B37] Schmechel D. E. Saunders A. M. Strittmatter W. J. Crain B. J. Hulette C. M. Joo S. H. (1993). Increased amyloid beta-peptide deposition in cerebral cortex as a consequence of apolipoprotein E genotype in late-onset Alzheimer disease. *Proc. Natl. Acad. Sci. U.S.A.* 90 9649–9653. 10.1073/pnas.90.20.9649 8415756 PMC47627

[B38] Schumacher J. Peraza L. R. Firbank M. Thomas A. J. Kaiser M. Gallagher P. (2019). Dynamic functional connectivity changes in dementia with Lewy bodies and Alzheimer’s disease. *Neuroimage Clin.* 22:101812. 10.1016/j.nicl.2019.101812 30991620 PMC6462776

[B39] Seeley W. W. Menon V. Schatzberg A. F. Keller J. Glover G. H. Kenna H. (2007). Dissociable intrinsic connectivity networks for salience processing and executive control. *J. Neurosci.* 27 2349–2356. 10.1523/JNEUROSCI.5587-06.2007 17329432 PMC2680293

[B40] Sperling R. A. Aisen P. S. Beckett L. A. Bennett D. A. Craft S. Fagan A. M. (2011). Toward defining the preclinical stages of Alzheimer’s disease: recommendations from the National Institute on Aging Alzheimer’s association workgroups on diagnostic guidelines for Alzheimer’s disease. *Alzheimers Dement.* 7 280–292. 10.1016/j.jalz.2011.03.003 21514248 PMC3220946

[B41] Sun Y. Dai Z. Li Y. Sheng C. Li H. Wang X. (2016). Subjective cognitive decline mapping functional and structural brain changes a combined resting-state functional and structural MR imaging study. *Radiology* 281 185–192. 10.1148/radiol.2016151771 27002419

[B42] Vaz A. P. Inati S. K. Brunel N. Zaghloul K. A. (2019). Coupled ripple oscillations between the medial temporal lobe and neocortex retrieve human memory. *Science* 363 975–978. 10.1126/science.aau8956 30819961 PMC6478623

[B43] Viviano R. P. Damoiseaux J. S. (2020). Functional neuroimaging in subjective cognitive decline: current status and a research path forward. *Alzheimers Res. Ther.* 12:23. 10.1186/s13195-020-00591-9 32151277 PMC7063727

[B44] Xie Y. Liu T. Ai J. Chen D. Zhuo Y. Zhao G. (2019). Changes in centrality frequency of the default mode network in individuals with subjective cognitive decline. *Front. Aging Neurosci.* 11:118. 10.3389/fnagi.2019.00118 31281248 PMC6595963

[B45] Xue C. Sun H. Hu G. Qi W. Yue Y. Rao J. (2020). Disrupted patterns of rich-club and diverse-club organizations in subjective cognitive decline and amnestic mild cognitive impairment. *Front. Neurosci.* 14:575652. 10.3389/fnins.2020.575652 33177982 PMC7593791

[B46] Zalesky A. Fornito A. Bullmore E. T. (2010). Network-based statistic: identifying differences in brain networks. *Neuroimage* 53 1197–1207. 10.1016/j.neuroimage.2010.06.041 20600983

