# Supplementary material for: Dynamics of the Brain Functional Network Associated With Subjective Cognitive Decline and Its Relationship to Apolipoprotein E €4 Alleles
Source: Front Aging Neurosci. 2022 Mar 9;14:806032. doi: 10.3389/fnagi.2022.806032 (PMC8959928; doi:10.3389/fnagi.2022.806032)
Supplement: Supplementary file 1 [file Data_Sheet_1.docx]

**Supplementary methods**

The GRETNA toolbox (<http://www.nitrc.org/projects/gretna/>) was used to calculate network properties of brain networks. We applied a wide range of sparsity (S) thresholds to all correlation matrices. The value of S was chosen to ensure that thresholded networks were estimable for the small-worldness scalar and the small-world index (σ) was larger than 1.0 (Watts & Strogatz, 1998). The range of our S thresholds was set to 0.10 < S < 0.34 with an interval of 0.01. For each network metric, the area under the curve (AUC) was calculated, which provides a summarized scalar for the topological characterization of brain networks independent of a single threshold selection. The AUC metric has proven to be sensitive for the detection of topological alterations of brain networks. Two network efficiency parameters (Latora & Marchiori, 2001), including the local efficiency (Eloc) and the global efficiency (Eglob) as well as two nodal properties, nodal degree (Rubinov & Sporns, 2010) and nodal efficiency (Achard & Bullmore, 2007), were finally computed.

The network-based statistic (NBS) was calculated by the methodology reported by previous study (Zalesky, Fornito, & Bullmore, 2010) with the family-wise error rate (FWER) correction (p ＜ 0.05) and 5000 times permutations was also performed.

***Referrence***

Achard, S., & Bullmore, E. (2007). Efficiency and cost of economical brain functional networks. *PLoS Comput Biol, 3*(2), e17. doi:10.1371/journal.pcbi.0030017

Latora, V., & Marchiori, M. (2001). Efficient behavior of small-world networks. *Phys Rev Lett, 87*(19), 198701. doi:10.1103/PhysRevLett.87.198701

Rubinov, M., & Sporns, O. (2010). Complex network measures of brain connectivity: uses and interpretations. *Neuroimage, 52*(3), 1059-1069. doi:10.1016/j.neuroimage.2009.10.003

Watts, D. J., & Strogatz, S. H. (1998). Collective dynamics of 'small-world' networks. *Nature, 393*(6684), 440-442. doi:10.1038/30918

Zalesky, A., Fornito, A., & Bullmore, E. T. (2010). Network-based statistic: identifying differences in brain networks. *Neuroimage, 53*(4), 1197-1207. doi:10.1016/j.neuroimage.2010.06.041

**Supplementary data**

Supplementary Table 1. The statistical significance at all cluster levels between k = 3 and k = 12 for life time of each state for the comparison between subjective cognitive decline patients and healthy controls. For each clustering level (k in each line) and state (column) we indicate the FDR corrected statistical significance.

| **P** | S1 | S2 | S3 | S4 | S5 | S6 | S7 | S8 | S9 | S10 | S11 | S12 |
| --- | --- | --- | --- | --- | --- | --- | --- | --- | --- | --- | --- | --- |
| k=3 | 0.247 | 0.302 | 0.382 | N/A | N/A | N/A | N/A | N/A | N/A | N/A | N/A | N/A |
| k=4 | 0.391 | 0.224 | 0.003 | 0.172 | N/A | N/A | N/A | N/A | N/A | N/A | N/A | N/A |
| k=5 | 0.320 | 0.431 | 0.061 | 0.015 | 0.043 | N/A | N/A | N/A | N/A | N/A | N/A | N/A |
| k=6 | 0.120 | 0.334 | 0.051 | 0.124 | 0.028 | 0.159 | N/A | N/A | N/A | N/A | N/A | N/A |
| k=7 | 0.035 | 0.145 | 0.367 | 0.020 | 0.340 | 0.294 | 0.162 | N/A | N/A | N/A | N/A | N/A |
| k=8 | 0.272 | 0.375 | 0.035 | 0.232 | 0.015 | 0.322 | 0.181 | 0.388 | N/A | N/A | N/A | N/A |
| k=9 | 0.286 | 0.011 | 0.225 | 0.354 | 0.275 | 0.181 | 0.476 | 0.034 | 0.065 | N/A | N/A | N/A |
| k=10 | 0.278 | 0.257 | 0.124 | 0.343 | 0.358 | 0.081 | 0.056 | 0.207 | 0.034 | 0.407 | N/A | N/A |
| k=11 | 0.213 | 0.122 | 0.292 | 0.427 | 0.102 | 0.042 | 0.274 | 0.369 | 0.295 | 0.354 | 0.125 | N/A |
| k=12 | 0.334 | 0.130 | 0.304 | 0.175 | 0.245 | 0.264 | 0.043 | 0.046 | 0.398 | 0.080 | 0.452 | 0.306 |

Supplementary Table 2. The statistical significance at all cluster levels between k = 3 and k = 12 for probability of each state for the comparison between subjective cognitive decline patients and healthy controls. For each clustering level (k in each line) and state (column) we indicate the FDR corrected statistical significance.

| **P** | S1 | S2 | S3 | S4 | S5 | S6 | S7 | S8 | S9 | S10 | S11 | S12 |
| --- | --- | --- | --- | --- | --- | --- | --- | --- | --- | --- | --- | --- |
| k=3 | 0.195 | 0.180 | 0.472 | N/A | N/A | N/A | N/A | N/A | N/A | N/A | N/A | N/A |
| k=4 | 0.388 | 0.073 | 0.009 | 0.387 | N/A | N/A | N/A | N/A | N/A | N/A | N/A | N/A |
| k=5 | 0.165 | 0.259 | 0.229 | 0.039 | 0.208 | N/A | N/A | N/A | N/A | N/A | N/A | N/A |
| k=6 | 0.145 | 0.160 | 0.067 | 0.368 | 0.255 | 0.301 | N/A | N/A | N/A | N/A | N/A | N/A |
| k=7 | 0.095 | 0.250 | 0.182 | 0.246 | 0.140 | 0.148 | 0.153 | N/A | N/A | N/A | N/A | N/A |
| k=8 | 0.211 | 0.098 | 0.079 | 0.250 | 0.344 | 0.434 | 0.171 | 0.142 | N/A | N/A | N/A | N/A |
| k=9 | 0.455 | 0.392 | 0.145 | 0.498 | 0.192 | 0.196 | 0.139 | 0.064 | 0.258 | N/A | N/A | N/A |
| k=10 | 0.208 | 0.207 | 0.076 | 0.051 | 0.380 | 0.391 | 0.187 | 0.176 | 0.016 | 0.335 | N/A | N/A |
| k=11 | 0.182 | 0.269 | 0.084 | 0.283 | 0.152 | 0.014 | 0.063 | 0.237 | 0.484 | 0.156 | 0.414 | N/A |
| k=12 | 0.425 | 0.297 | 0.164 | 0.331 | 0.088 | 0.433 | 0.105 | 0.010 | 0.153 | 0.361 | 0.122 | 0.368 |

Supplementary Table 3. The leading eigenvector for each PL state when k = 4.

|  | State 1 | State 2 | State 3 | State 4 |
| --- | --- | --- | --- | --- |
| Region names | Hippocampus.R | Precentral.L | Precentral.L | Frontal_Sup.L |
|  | ParaHippocampal.R | Precentral.R | Precentral.R | Frontal_Sup.R |
|  | Calcarine.L | Frontal_Sup.L | Rolandic_Oper.L | ORBsup.L |
|  | Calcarine.R | Frontal_Sup.R | Rolandic_Oper.R | ORBsup.R |
|  | Cuneus.L | Frontal_Mid.L | Insular.L | Frontal_Mid.L |
|  | Cuneus.R | Frontal_Mid.R | Insular.R | Frontal_Mid.R |
|  | Lingual.L | IFGoperc.L | Hippocampus.R | ORBmid.L |
|  | Lingual.R | IFGoperc.R | ParaHippocampal.R | ORBmid.R |
|  | Occipital_Sup.L | IFGtriang.L | Calcarine.L | IFGtriang.L |
|  | Occipital_Sup.R | IFGtriang.R | Calcarine.R | ORBinf.L |
|  | Occipital_Mid.L | Rolandic_Oper.L | Cuneus.L | ORBinf.R |
|  | Occipital_Mid.R | Rolandic_Oper.R | Cuneus.R | Supp_Motor_Area.L |
|  | Occipital_Inf.L | Supp_Motor_Area.L | Lingual.L | SFGmed.L |
|  | Occipital_Inf.R | Supp_Motor_Area.R | Lingual.R | SFGmed.R |
|  | Fusiform.L | Cingulum_Mid.L | Occipital_Sup.L | ORBsupmed.L |
|  | Fusiform.R | Cingulum_Mid.R | Occipital_Sup.R | ORBsupmed.R |
|  | Parietal_Sup.L | Postcentral.L | Occipital_Mid.L | Rectus.L |
|  | Parietal_Sup.R | Postcentral.R | Occipital_Mid.R | Rectus.R |
|  | Precuneus.L | Parietal_Sup.L | Occipital_Inf.L | Cingulum_Ant.L |
|  | Precuneus.R | Parietal_Sup.R | Occipital_Inf.R | Cingulum_Ant.R |
|  | Temporal_Inf.L | Parietal_Inf.L | Fusiform.L | Cingulum_Post.L |
|  | Temporal_Inf.R | Parietal_Inf.R | Fusiform.R | Cingulum_Post.R |
|  |  | SupraMarginal.L | Postcentral.L | Angular.L |
|  |  | SupraMarginal.R | Postcentral.R | Angular.R |
|  |  | Angular.R | Parietal_Sup.L | Caudate.L |
|  |  | Precuneus.L | Parietal_Sup.R | Caudate.R |
|  |  | Precuneus.R | SupraMarginal.L | THA.L |
|  |  | Paracentral_Lobule.L | SupraMarginal.R | THA.R |
|  |  | Paracentral_Lobule.R | Paracentral_Lobule.L | Temporal_Inf.L |
|  |  |  | Paracentral_Lobule.R | Temporal_Inf.R |
|  |  |  | Heschl.L |  |
|  |  |  | Heschl.R |  |
|  |  |  | Temporal_Sup.L |  |
|  |  |  | Temporal_Sup.R |  |
|  |  |  | Temporal_Pole_Sup.L |  |
|  |  |  | Temporal_Pole_Sup.R |  |
|  |  |  | Temporal_Mid.R |  |
